# Simultaneous quantitative assessment of circulating cell-free mitochondrial and nuclear DNA by multiplex real-time PCR

**DOI:** 10.1590/S1415-47572009000100003

**Published:** 2009-03-01

**Authors:** Peng Xia, Ramin Radpour, Rebecca Zachariah, Alex Xiu Cheng Fan, Corina Kohler, Sinuhe Hahn, Wolfgang Holzgreve, Xiao Yan Zhong

**Affiliations:** 1 Laboratory for Prenatal Medicine and Gynaecological Oncology, Women's Hospital, University of BaselSwitzerland; 2 Department of Oncology Surgery, First Affiliated Hospital of the Medical College, Xi'an Jiaotong University, Xi'anChina; 3 , University Medical Center FreiburgGermany

**Keywords:** circulating cell-free DNA, mitochondrial DNA, nuclear DNA, real-time PCR, quantitative PCR

## Abstract

Quantification of circulating nucleic acids in plasma and serum could be used as a non-invasive diagnostic tool for monitoring a wide variety of diseases and conditions. We describe here a rapid, simple and accurate multiplex real-time PCR method for direct synchronized analysis of circulating cell-free (ccf) mitochondrial (mtDNA) and nuclear (nDNA) DNA in plasma and serum samples. The method is based on one-step multiplex real-time PCR using a FAM-labeled MGB probe and primers to amplify the mtDNA sequence of the ATP 8 gene, and a VIC-labeled MGB probe and primers to amplify the nDNA sequence of the glycerinaldehyde-3-phosphate-dehydrogenase (GAPDH) gene, in plasma and serum samples simultaneously. The efficiencies of the multiplex assays were measured in serial dilutions. Based on the simulation of the PCR reaction kinetics, the relative quantities of ccf mtDNA were calculated using a very simple equation. Using our optimised real-time PCR conditions, close to 100% efficiency was obtained from the two assays. The two assays performed in the dilution series showed very good and reproducible correlation to each other. This optimised multiplex real-time PCR protocol can be widely used for synchronized quantification of mtDNA and nDNA in different samples, with a very high rate of efficiency.

## Introduction

Eukaryotic cells have nuclear DNA (nDNA) and additional cytoplasmic mitochondrial DNA (mtDNA). It has been demonstrated that cell-free nucleic acids, *i.e.*, cell-free (ccf) nuclear DNA (cf-nDNA) and ccf mtDNA exist in circulation (Sozzi *et al.*, 2003). Quantification of ccf nDNA and ccf mtDNA concentrations in plasma and serum has raised great interest as a tool for non-invasive diagnosis and monitoring of a wide variety of diseases and conditions, such as cancers (Zhong *et al.*, 2007c), pathological pregnancies (Zhong *et al.*, 2001), inflammatory disease (Zhong *et al.*, 2007d) and trauma (Lam *et al.*, 2003). It has been reported that both circulating plasma nDNA and mtDNA were increased after trauma (Lam *et al.*, 2003; Lam *et al.*, 2004). Many studies observed elevated levels of ccf nDNA in plasma or serum of various cancers (Allen *et al.*, 2004; Gormally *et al.*, 2004; Sozzi *et al.*, 2003; Taback *et al.*, 2004). Elevated levels of mtDNA were detected in plasma of prostate cancer patients using quantitative real-time PCR amplification (Mehra *et al.*, 2007). Recently, Ellinger *et al.* (2008) observed that mtDNA in serum of patients with prostate cancer has a predictive value of biochemical recurrence after prostatectomy. The observations suggested that ccf DNA might be a potentially valuable prognostic marker for these patients. The similar increase of ccf mtDNA and ccf nDNA in cancers, and in patients after trauma, implies that both the nDNA and mtDNA might be released from the same tissues of origin and by similar mechanisms. Since the total amount of mtDNA per cell is unknown, determining both species in a single reaction would be the most effective and accurate method to compare relative mtDNA quantities with nDNA genome equivalents. Furthermore, the multiplexed assay for simultaneous testing of two parameters can reduce the time consumed by the diagnostic procedures.

In our study, we developed a rapid, simple and accurate multiplex real-time PCR method for direct synchronized analysis of mtDNA and nDNA in paired plasma and serum samples. This method is based on a single-step real-time PCR, using a FAM- and a VIC-labelled probe for determining selected mtDNA and nDNA regions of interest. We optimised the multiplex assays for amplifying nDNA and mtDNA simultaneously and efficiently. Since most methods for DNA extraction are established for extracting and purifying nDNA, in our study we also compared the mtDNA and nDNA quantities by using three commercial kits for ccf DNA extraction.

## Materials and Methods

### Sample collection

Paired plasma and serum samples were obtained from 25 healthy blood donors, with informed consent. The study was approved by the local institutional review board.

### Processing of blood samples

The 10 mL of peripheral blood samples for coagulant serum and 10 mL of peripheral blood samples for EDTA plasma were taken from blood donors. The blood samples were processed immediately by centrifugation at 1600 g for 10 min. The plasma and serum layers were transferred to new Eppendorf tubes and centrifuged again at maximum speed (16000 g) for 10 min. Plasma and serum samples were divided into aliquots of 400 μL each and stored at -80 °C.

### DNA extraction

Since there are no commercial kits for ccf mtDNA extraction from serum and plasma, we firstly compared three different DNA extraction methods for co-extracting nDNA and mtDNA using plasma and serum samples from five individuals. DNA extraction from the five paired serum and plasma samples was performed using the QIAamp DNA mini kit (QIAGEN) for a first aliquot. For a second aliquot, we used the High pure PCR template preparation kit (Roche Applied Science), and for a third aliquot the automated method with the MagNA Pure LC DNA Isolation Kit – large Volume (Roche Applied Science) and the MagNA Pure LC Instrument. Visually, the automated method with MagNA Pure LC DNA Isolation Kit seemed to yield larger amounts of mtDNA and nDNA, however no significant differences were observed in the quantities using the different commercial kits (Kruskal-Wallis-Test: p = 0.32 for nDNA; and p = 0.194 for mtDNA, respectively). Using the automated method, ccf DNA was extracted from each 400 μL plasma and serum sample, and the DNA preparations were eluted in 100 μL elution buffer according to the MagNA Pure LC software.

### Quantitative analysis of ccf DNA in plasma and serum samples

Five μL of DNA elution were used as template for the real-time PCR analysis. For testing nDNA, the GAPDH housekeeping gene was used with forward 5'-CCCCAC ACACATGCACTTACC-3' and reverse 5'-CCTAGTCCC AGGGCTTTGATT-3' primers and 5'-MGB-TAGGAAG GACAGGCAAC – VIC-3' as the probe. For determining mtDNA, a sequence of the MTATP 8 gene starting at locus 8446 was amplified, with forward primer 5'-AATATTAA ACACAAACTACCACCTACC-3', reverse primer 5'-TGGTTCTCAGGGTTTGTTATAA-3' and a 5'-6-FAM-CCTCACCAAAGCCCATA-MGB-3' probe (Walker *et al.*, 2005). PCR was performed using an ABI PRISM 7000 Sequence Detection System (Applied Biosystems, ABI) in a total reaction volume of 25 μL, containing 5 μL of DNA, 12.5 μL of TaqMan® Universal PCR Master Mix, 4 primers and 2 probes, using a 2 min incubation at 50 °C, followed by an initial denaturation step at 95 °C for 10 min and 40 cycles of 1 min at 60 °C and 15 s at 95 °C. For the simultaneous multiplex TaqMan amplification of the two species, we optimised the concentration of primers and probes, which were: 0.6 μM for each primer and 0.4 μM for each probe.

### Efficiency Measurements of the multiplex assays

The efficiency of the multiplex assay for amplifying both nDNA and mtDNA was measured with standard curves generated by dilution series. Two kinds of dilution series were used for the measurements: 1) HPLC-purified single-stranded synthetic DNA oligonucleotides (Microsynth) specifying a 79-bp mtDNA amplicon and a 97 GAPDH amplicon with 6 concentration points ranging from 5 x 10^7^ copies to 5 x 10^2^ copies; 2) a known concentration of human genomic DNA with six points ranging from 3.125 x 10^4^ to 10 pg/μL (including 31250, 6250, 1250, 250, 50 and 10 pg/μL). The latter dilution series showed higher reproducible standard dilution curves than the former, and was therefore used for the further experiments.

### Quantitative assessment of ccf mtDNA and nDNA

The concentrations of ccf nDNA were estimated according to the standard curves, using the known concentration of human genomic DNA, and were expressed as genome-equivalents (GE) per mL of plasma or serum. A conversion factor of 6.6 pg of DNA per cell was used to calculate the GE (Garcia Moreira *et al.*, 2006), as shown in our previous studies on ccf nDNA (Zhong *et al.*, 2007a; Zhong *et al.*, 2007b; Zhong *et al.*, 2007c). Fold change of ccf mtDNA could be calculated using two methods (Liu and Saint, 2002):
1) 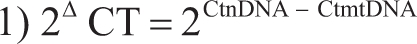

2) 
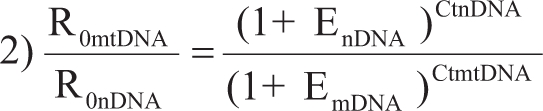


Relative quantities of ccf mtDNA could be estimated using an equation of GE_nDNA_ x fold-change_mtDNA_ and expressed also as GE per mL of plasma or serum.

### Statistical analysis

Data were analysed using the SPSS software (Statistical Software Package for Windows v. 15.0). Quantities of ccf mtDNA and ccf nDNA are expressed as median, range and fold change. The Spearman Rank Test was applied to analyse the relationship between mtDNA and nDNA amplifications. Mann-Whitney and Krurkal-Wallis tests were used to determine the statistical significance of the differences between the measured concentrations of nDNA and mtDNA.

## Results

### Optimised experimental design and conditions for the multiplex assays

Ccf DNA extracted by two different manual methods and one automated method was amplified and compared for differences in the quantification of nDNA and mtDNA. Using the three different kits, it was possible to co-extract nDNA and mtDNA from plasma and serum samples. The automated method showed greater advantages, as it proved less time- and labour-consuming, and minimized the risk of contamination, and was therefore used for further experiments.

The primers and probes for amplifying GAPDH have been successfully used in our many previous studies, and the specificity of the assay has been confirmed (Lapaire *et al.*, 2007; Zanetti-Dallenbach *et al.*, 2007). To assess the specificity of the assay for amplifying mtDNA, the ρ0 cell line without mtDNA was tested. There was no false-positive amplification for mtDNA in the ρ0 cells observed (Xiu-Cheng Fan *et al.*, 2008).

### Amplification efficiencies of the multiplex assays

**Figure 1:**
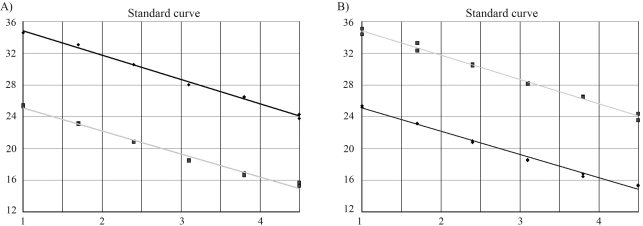
Simulation of real-time PCR kinetics for amplifying mtDNA and nDNA on serial dilutions. The figure shows reproducible standard dilution curves for identification of the mtDNA and nDNA. The upper lines are nDNA standard dilution curves and the lower lines are mtDNA standard dilution curves. The numbers on the y axis represent the values of cycle threshold (Ct) and numbers on the x axis represent the dilution points.

We analysed 10 standard curves, using a known concentration of human genomic DNA containing six concentration points for both mtDNA and nDNA. The standard curves with average slopes at approximately -3.3 (~100% efficiency) were obtained using our optimised TaqMan PCR conditions. The two assays on the dilution series were very similar and showed very good correlation to each other and reproducibility. The average correlation coefficient of the 10 standard curves using the Spearman Rank Test was 0.99 (range: 0.989-0.999, p < 0.001) Figure 1 shows two examples of the standard curves with a correlation coefficient of 0.994 and 0.997, respectively.

### Quantitative assessment of ccf nDNA and ccf mtDNA in serum and plasma

The ccf nDNA equivalents were calculated according to the standardised method, using very reproducible standard dilution curves, which have been described in our previous studies (Lapaire *et al.*, 2007; Zanetti-Dallenbach *et al.*, 2007; Zhong *et al.*, 2007a). Based on the comparative amplifications of nDNA and mtDNA with an efficiency close to 100%, the fold changes of ccf mtDNA were calculated using the equation of 2^CtnDNA-CtmtDNA^ (R_0mtDNA_ / R_0nDNA_ = (1 + E_nDNA_)^CtnDNA^ / (1 + E_mtDNA_)^CtmtDNA^ = (1 + E_nDNA_)^CtnDNA-^^CtmtDNA,^ if E_nDNA_ = E_mtDNA_; (1 + E_nDNA_)^CtnDNA-^^CtmtDNA^ 2^CtnDNA-CtmtDNA^, if E close to 100%).

The relative equivalents of ccf mtDNA were estimated, and both the quantities of ccf nDNA and ccf mtDNA in the 20 paired plasma and serum samples are shown in Table 1.

Ccf mtDNA and nDNA were determined by multiplex real-time PCR, with a mean concentration of 208,184 GE/mL and 6,106 GE/mL in the plasma samples, respectively, and of 2,491,364 GE/mL and 273,337 GE/mL in the serum samples, respectively. The ccf nDNA and ccf mtDNA levels in the serum samples were significantly higher than those of the plasma samples (12-45 fold). This may be a result of the release of cellular DNA artefacts during blood clotting procedures. The ccf mtDNA levels in the plasma and serum samples were significantly higher than those of ccf nDNA (9-34 fold).

## Discussion

In this study, we described the multiplex assays used to analyse ccf nDNA and ccf mtDNA simultaneously. Using the optimised PCR protocol, the amplification of nDNA and mtDNA in a single reaction tube was very similar and showed a simulation of real-time PCR kinetics. With the comparative efficiencies of two assays in a single tube, we could use the nDNA level as a reference to assess the relative quantities of mtDNA. The method is simple and does not require generating standard curves, which often result in errors due to dilution inaccuracy. Using the multiplex assays, we could rapidly and accurately determine the levels of ccf nDNA and ccf mtDNA in serum and plasma samples. The levels of ccf mtDNA in circulation are significantly higher than those of ccf nDNA, due to the fact that the number of mitochondrial genomes in a cell ranges from several hundreds to more than 10,000 copies, and each mitochondrion contains between two and 10 mtDNA molecules (Higuchi, 2007). The serum samples showed a significantly higher concentration of ccf DNA because of cellular DNA release during the blood clotting procedures (Zanetti-Dallenbach, 2008; Zhong *et al.*, 2007a).

Quantitative alterations of ccf nDNA and mtDNA have been observed in many conditions, especially in aging, apoptosis and carcinogenesis (Goebel *et al.*, 2005; Liu *et al.*, 2003; Mehra *et al.*, 2007; Takeuchi *et al.*, 2004). So far, the exact content of human mtDNA in different cells and tissues remains unclear. Two studies developed multiplex assays to analyse nDNA and mtDNA simultaneously for forensic medicine (Alonso *et al.*, 2004; Walker *et al.*, 2005). In our study, we were able to use the GAPDH gene as a housekeeping gene to analyse the quantities of mtDNA. The aim of this study was to develop a rapid, simple and accurate multiplex real-time PCR for the direct synchronized analysis of ccf mtDNA and ccf nDNA, which may provide a platform for further investigations leading to a better understanding of the biology of ccf mtDNA and ccf nDNA on large-scale sample sizes.

It is known that ancient mtDNA sequences, also termed as nuclear pseudogenes, are present in the human nuclear genome as multiple copies. Woischnik and Moraes (2002) found up to 612 nuclear integrations. Their homology with the current mtDNA was up to 99%. An accidental co-amplification of these nuclear copies of mitochondrial genes might bias the results (Wallace *et al.*, 1997). We tested the specificity by using the mitochondria-negative cell line ρ0, and no mtDNA signals were detected in this cell line by real-time multiplex PCR.

Based on the importance of quantification of both mtDNA and nDNA in life science, we developed a rapid, accurate, simple and low-cost approach that enables the simultaneous identification of physiological and pathogenic mtDNA and nDNA variants. Since there are no commercial kits for ccf mtDNA extraction from serum and plasma samples, we, for the first time, compared three different DNA extraction methods for co-extracting nDNA and mtDNA from plasma and serum. We were also the first ones to examine the efficiencies of the two assays in a single tube. After calculating the comparative efficiencies we could use the nDNA level as a reference to assess the relative quantities of mtDNA, which can simplify the calculation of mtDNA content. The method is simple and does not require generating standard curves, which often result in errors due to dilution inaccuracy. This method can be considered a standard approach for widely quantifying both mtDNA and nDNA in different kinds of samples.

## Figures and Tables

**Table 1:** Ccf mtDNA and nDNA represented in genome equivalent (GE)/mL in serum and plasma

	mtDNA	nDNA	mtDNA/nDNA	p- value
Serum	2491364 (33787-11720914)	273337 (14845-1426767)	9 fold	< 0.001
Plasma	208184 (8027-2618508)	6106 (465-61722)	34 fold	< 0.001
Serum/plasma	12 fold	45 fold		
p-value	< 0.001	< 0.001		
